# Multimorbidity and Mental Health Trajectories Among Middle-Aged and Older U.S. Adults During the COVID-19 Pandemic: Longitudinal Findings From the COVID-19 Coping Study

**DOI:** 10.1093/geroni/igac047

**Published:** 2022-07-30

**Authors:** Greta Jianjia Cheng, Abram L Wagner, Brendan Q O’Shea, Carly A Joseph, Jessica M Finlay, Lindsay C Kobayashi

**Affiliations:** Department of Epidemiology, University of Michigan School of Public Health, Ann Arbor, Michigan, USA; Brain, Environment, Aging, and Mobility (BEAM) Lab, Department of Epidemiology, University of Pittsburgh School of Public Health, Pittsburgh, PA, USA; Department of Epidemiology, University of Michigan School of Public Health, Ann Arbor, Michigan, USA; Department of Epidemiology, University of Michigan School of Public Health, Ann Arbor, Michigan, USA; Department of Epidemiology, University of Michigan School of Public Health, Ann Arbor, Michigan, USA; Social Environment and Health Program, Institute for Social Research, University of Michigan, Ann Arbor, Michigan, USA; Department of Epidemiology, University of Michigan School of Public Health, Ann Arbor, Michigan, USA

**Keywords:** Depression and anxiety, Loneliness, Multiple chronic conditions

## Abstract

**Background and Objectives:**

This study aimed to examine the associations between multimorbidity at the COVID-19 pandemic onset and subsequent longitudinal trajectories of depressive symptoms, anxiety symptoms, and loneliness in middle-aged and older adults over a 12-month follow-up.

**Research Design and Methods:**

Data were from monthly online questionnaires in the COVID-19 Coping Study of U.S. adults aged ≥55 from April/May 2020 through April/May 2021 (*N* = 4,024). Multimorbidity was defined as having ≥2 versus <2 chronic conditions at baseline. Mental health outcomes were assessed monthly as depressive symptoms (8-item Center for Epidemiologic Studies Depression scale), anxiety symptoms (5-item Beck Anxiety Inventory), and loneliness (3-item UCLA Loneliness Scale). We used multivariable-adjusted population- and attrition-weighted mixed-effects linear models to examine the longitudinal associations between multimorbidity and mental health symptoms.

**Results:**

Multimorbidity at the pandemic onset was associated with elevated depressive (*b* = 0.37; 95% CI: 0.16–0.59) and anxiety (*b* = 0.39; 95% CI: 0.15–0.62) symptoms at baseline. Changes in symptoms for all three mental health outcomes were nonlinear over time, with worsening symptoms over the first 6 months of the pandemic (April/May to September/October 2020), followed by improvement in symptoms over the subsequent 6 months (September/October 2020 to April/May 2021). Middle-aged and older adults with multimorbidity experienced faster rates of change in anxiety symptoms and loneliness than those without multimorbidity, with persistently elevated mental health symptomatology throughout the follow-up.

**Discussion and Implications:**

Results highlight the unique and persistent mental health risks experienced by middle-aged and older adults with multimorbidity during the COVID-19 pandemic. The observed improvements in symptoms underscore the mental resilience of these individuals, indicating their adaptation to the ongoing pandemic.

**Translational Significance:** This study examined the associations between multimorbidity at the COVID-19 pandemic onset and subsequent longitudinal trajectories of depressive symptoms, anxiety symptoms, and loneliness in U.S. adults aged ≥55 years over a 12-month follow-up. We found that middle-aged and older adults with multimorbidity measured at the pandemic onset had persistently higher depressive symptoms, anxiety symptoms, and loneliness throughout the subsequent 12 months than those without multimorbidity. Findings suggest that enhanced mental health screening and intervention strategies that target improving social connections and resilience can potentially lessen the mental health burden experienced by middle-aged and older adults with multimorbidity.

Multimorbidity is commonly defined as the coexistence of two or more chronic conditions (U.S. Department of Health and Human Services, 2010). In 2018, the prevalence of multimorbidity in United States adults was 27.2%, with a disproportionate burden among middle-aged and older adults (33% among those aged 45 to 64 years and 64% among those aged 65+; Boersma et al., 2020). Multimorbidity in middle-aged and older populations has been prospectively linked to incident frailty (Vetrano et al., 2019), functional and cognitive decline (Wei & Mukama, 2018; Wei et al., 2020), psychosocial distress (Fortin, 2006; Gunn et al., 2012; Jones et al., 2016; Parajuli et al., 2021), and all-cause mortality (Wei & Mukama, 2018). Along with adverse clinical outcomes, individuals with multimorbidity commonly experience increased use of health care, poor coordination of care, polypharmacy, greater treatment burden, and burden related to disease self-management (Moffat & Mercer, 2015; Wallace et al., 2015).

Older adults with multimorbidity may be particularly vulnerable to health risks during the coronavirus disease 2019 (COVID-19) pandemic. Underlying chronic conditions position them at elevated risk for COVID-19 related complications, hospitalization, and death due to compromised immune system function (Bialek et al., 2020). Furthermore, as COVID-19 cases surged across the United States in multiple waves in 2020, there were delays in delivering care to non-COVID-19 patients with preexisting chronic conditions, and cancelations of routine medical care, screening visits, laboratory monitoring visits, and closure of physical therapy facilities (Czeisler et al., 2020). Limited access to routine health care and home care visits, as well as avoidance of health care facilities due to the fear of infection, may exacerbate the onset and the progression of chronic conditions. Older adults with multimorbidity may also have been particularly socially isolated during the pandemic, in order to minimize their COVID-19 risk (Gorenko et al., 2021). Based on stress and coping theories (Lazarus & Folkman, 1984), negative expectations surrounding the pandemic such as worries about the risks of infection, severe illness, hospitalization, mortality, disruption of health services, and greater social isolation may have led to negative mental health effects for older adults with multimorbidity (Davis et al., 2021; Pearman et al., 2021; Whitehead, 2021). However, there is a paucity of research examining the mental health trajectories of middle-aged and older adults with multimorbidity during the COVID-19 pandemic.

Several surveys and some longitudinal studies during the COVID-19 pandemic indicate that the general population of middle-aged and older adults has experienced increasing challenges in mental health since the onset of the COVID-19 pandemic, manifested in increased worries, depressive symptoms, anxiety symptoms, and loneliness (Fancourt et al., 2021; Fluharty et al., 2021; Luchetti et al., 2020; Mooldijk et al., 2022; Raina et al., 2021; van Tilburg et al., 2021; Wright et al., 2021; Zaninotto et al., 2022). Studies conducted prior to the COVID-19 pandemic have identified a dose–response relationship between number of chronic conditions and psychological symptom burden in adult populations, including older adults (Cheng et al., 2021; Fortin, 2006; Gunn et al., 2012; Jones et al., 2016; Parajuli et al., 2021; Xiang & Cheng, 2019). In a cross-sectional study of 7,026 U.S. older adults aged ≥65 from the National Health and Aging Trends Study in 2011, Jones et al. (2016) found that a greater number of chronic conditions was associated with higher levels of depressive and anxiety symptoms after adjusting for sociodemographic and health-related characteristics. However, pre-pandemic evidence on the association between multimorbidity and psychosocial distress has been largely restricted to cross-sectional designs (Fortin, 2006; Gunn et al., 2012; Jones et al., 2016; Parajuli et al., 2021). Limited longitudinal studies have considered the trajectories of mental health symptoms stratified by multimorbidity status, either before or during the COVID-19 pandemic. Of those that examined the trajectories of mental health conditions prior to the pandemic, the increased number of chronic conditions was found to be a predictor of persistent and recurrent depressive and anxiety symptoms (Chen et al., 2012; Cheng et al., 2021; Shin & Cho, 2022; Xiang & Cheng, 2019).

It is unknown how middle-aged and older adults with multimorbidity have fared with respect to mental health compared to their counterparts without multiple chronic conditions during the pandemic. The current study aimed to estimate the associations between multimorbidity at the pandemic onset and subsequent longitudinal trajectories of depressive symptoms, anxiety symptoms, and loneliness in older adults over a 12-month follow-up. We hypothesized that, relative to those without multimorbidity, participants with multimorbidity would (a) report greater depressive symptoms, anxiety symptoms, and loneliness at the study baseline (April/May 2020) and (b) experience faster rates of change in depressive symptoms, anxiety symptoms, and loneliness in the subsequent 12 months, as their particular vulnerability to adverse COVID-19 outcomes, disrupted routine health care, and social isolation may predispose them to worse mental health outcomes during the pandemic period.

## Method

### Data and Sample

Data were from the COVID-19 Coping Study, a longitudinal, mixed-methods study of adults ≥55 years residing in all 50 U.S. states, the District of Columbia, and Puerto Rico (Kobayashi et al., 2021). The COVID-19 Coping Study aims to examine the mental health and well-being of middle-aged and older U.S. adults in relation to social, economic, and behavioral changes during the COVID-19 pandemic. Details of the sample, study design, recruitment, and methodology are published elsewhere (Kobayashi et al., 2021). In brief, a total of 6,938 participants were recruited using an online multiframe, nonprobability sampling strategy from April 2 through May 31, 2020. Of these, 4,401 were included in a longitudinal subsample who were invited to complete monthly follow-ups for a 12-month period following baseline, until April/May 2021. These 4,401 participants were eligible for the present analysis. Of the eligible participants, three were excluded because they were missing mental health outcome data at every follow-up wave and 374 were excluded because they were missing covariate data. The final analytical sample consisted of 4,024 participants (91% of eligible). The median follow-up time was 7 months (interquartile range: 4–10 months).

### Measures

#### Exposure: multimorbidity

At baseline, participants self-reported whether they had previously been diagnosed with each of the following conditions by a doctor: hypertension, diabetes, heart disease, asthma, chronic obstructive pulmonary disease, cancer, or another limiting, longstanding health condition. Multimorbidity was defined as the coexistence of two or more of these health conditions (<2 chronic conditions vs. ≥2 chronic conditions).

#### Outcomes: depressive symptoms, anxiety symptoms, loneliness

Depressive symptoms were measured at the baseline and at each of the 12 subsequent monthly follow-ups using the 8-item Center for Epidemiological Studies Depression scale (Cronbach’s alpha = .86), adapted from the U.S. Health and Retirement Study (Lewinsohn et al., 1997). Participants were asked if, in the past week, they felt (a) depressed; (b) that everything they did was an effort; (c) that their sleep was restless; (d) happy; (e) lonely; (f) that they enjoyed life; (g) sad; and (h) they could not get going. Response options for each item were “yes” (1 point) or “no” (0 points), with reverse coding for items (d) and (f). The total depressive symptom score was the sum of all responses, ranging from 0 to 8, with a higher score indicating higher levels of depressive symptoms.

Anxiety symptoms were measured at the baseline and at each of the 12 subsequent monthly follow-ups using the 5-item Beck Anxiety Scale (Cronbach’s alpha = .94), adapted from the U.S. Health and Retirement Study (Smith et al., 2013). Participants were asked how often in the past week they (a) had a fear of the worst happening; (b) felt nervous; (c) felt hands trembling; (d) had a fear of dying; and (e) felt faint. Response options were “never” (1 point), “hardly ever” (2 points), “some of the time” (3 points), and “most of the time” (4 points). The anxiety symptom score was the sum score of all responses, ranging from 4 to 20, with a higher score indicating higher levels of anxiety symptoms.

Loneliness was assessed at the baseline and at each of the 12 subsequent monthly follow-ups using the 3-item UCLA Loneliness Scale (Cronbach’s alpha = .89–.94; Russell, 1996), which asked participants how often in the past week they (a) felt they lacked companionship; (b) felt left out; (c) felt isolated from others. Response options were “hardly ever” (1 point), “some of the time” (2 points), and “often” (3 points). The loneliness score was the sum of all responses, ranging from 3 to 9, with a higher score indicating higher levels of loneliness.

#### Covariates

We included covariates that were potential confounders of the associations between multimorbidity and mental health outcomes: age (years); sex (male, female); race/ethnicity (non-Hispanic White, non-Hispanic Black, Hispanic/Latinx, non-Hispanic Other); educational attainment (high school or equivalent or less, some college or 2-year associate’s degree, 4-year college or university degree, graduate degree); partnership status (coupled, not coupled); pre-COVID-19 employment status (retired, employed full-time, employed part-time, self-employed, unable to work due to disability or health condition, homemaker or family caregiver, unemployed and seeking work); pre-COVID-19 social isolation (low, high), measured using the 5-point social isolation index from the English Longitudinal Study of Ageing (Kobayashi & Steptoe, 2018); previous diagnosis of depression (yes, no); previous diagnosis of anxiety (yes, no); use of any mobility aid (yes, no); smoking status (never smoked, ex-smoker, current smoker); pre-COVID-19 moderate-to-vigorous intensity exercise per week (none to 2.5 hr, in 30-min increments); and pre-COVID-19 alcohol consumption (number of drinks per week). All covariates were measured at baseline, except for preexisting depression and anxiety, which were assessed as self-reported diagnoses prior to April 2020 at the 2-month (May/June 2020) and 3-month (June/July 2020) follow-ups. Approximately 14% of the sample (*n* = 617/4,401) had missing values of preexisting diagnoses of depression and anxiety, as not all participants completed the 2- or 3-month follow-up surveys. We performed multiple imputation using chained equations to impute these missing values (Azur et al., 2011).

### Statistical Analyses

We described the sample characteristics overall and according to multimorbidity status. To account for potential selection and other nonsample biases due to the nonprobability nature of this sample, we applied population weights to all analyses. The population weight was generated based on age, sex, race/ethnicity, education, marital status, and U.S. census region of residence for the general U.S. population aged ≥55 years from the nationally representative 2018 American Community Survey (Kobayashi et al., 2021).

We specified multivariable-adjusted population- and attrition-weighted mixed-effects linear models with random person-specific intercepts and slopes using maximum likelihood estimation and independent covariance structure to estimate the associations between baseline multimorbidity status with levels of depressive symptoms, anxiety symptoms, and loneliness at the baseline (April/May 2020) and the rates of change in these mental health outcomes over a 12-month follow-up (April/May 2020 through April/May 2021). To account for potential nonlinear changes over time in the mental health outcomes, we included linear and quadratic terms for time in all models. We included interaction terms between linear time and multimorbidity, and quadratic time and multimorbidity, to evaluate whether the rates of change over time for each of depressive symptoms, anxiety symptoms, and loneliness varied by multimorbidity status measured at the pandemic onset. Our analytic model is denoted in the following equation ($b_{ij\;}and\;{\;\varepsilon }_{ij}\;$ represent random effects):


$$\begin{array}{l} \begin{array}{ll} & Mental\;health\;outcome_{ij}=\beta_{0}+\beta_{1}\;*\;multimorbidity\\ & \;\;\;\;+\;\beta_{2}\;*\;linear\;time+\beta_{3}\;*\;\left(multimorbidity\;\times \;linear\;time\right)\\ & \;\;\;\;+\;\beta_{4}\;*\;quadratic\;time\;\\ & \;\;\;\;+\;\beta_{5}\;*\;\left(multimorbidity\;\times \;quadratic\;time\;\right)\\ & \;\;\;\;+\;\beta_{6}\;*\;age+\beta_{7}\;*\;sex+\beta_{8}\;*\;race+\beta_{9}\;*\;education\\ & \;\;\;\;+\;\beta_{10}\;*\;partnership+\;\beta_{11}\;*\;employment\\ & \;\;\;\;+\;\beta_{12}\;*\;social\;isolation\;+\;\beta_{13}\;*\;smoking\;\;\;\\ & \;\;\;\;+\;\beta_{14}\;*\;exercise+\beta_{15}\;*\;drinking\\ & \;\;\;\;+\;\beta_{16}\;*\;depression\;diganosis+\;\beta_{17}\;\;anxiety\;diganosis\\ & \;\;\;\;+\;b_{0j}+\;b_{timej}+\varepsilon_{ij} \end{array} \end{array}$$

We used likelihood ratio tests to estimate the inclusion and exclusion of sets of covariates and interaction terms, and random slopes. We computed robust standard errors and all models incorporated the population weight at level 2 (person level) and the wave-specific weight at level 1 (observation level) within the multilevel models. Using methods detailed in Heeringa et al. (2017), we created the wave-specific weight for each respondent, accounting for the initial survey weight for each individual and differential attrition patterns at each wave. These wave-specific weights were also scaled so that their sum was equal to the available number of data points within each individual (Heeringa et al., 2017). All analyses were conducted using Stata 17.0 SE (College Station, TX) with a statistical significance level of .05.

## Results

Sample characteristics are given in Table 1. The mean age at baseline was 67.3 years (*SD*: 7.5 years, range: 55–99 years). The population-weighted prevalence of multimorbidity at baseline was 36.4% (95% CI: 34.2, 38.7). Details on the prevalence of the different types and counts of chronic conditions can be found in Supplementary Table 1.

**Table 1.** Population-Weighted Characteristics of Baseline Sample, COVID-19 Coping Study, United States, April/May 2020

Variable	Overall (unweighted *N* = 4,024)		Multimorbidity status			
			<2 chronic conditions (unweighted *n* = 2,720)		≥2 chronic conditions (unweighted *n* = 1,304)	
	%/Mean	95% CI	%/Mean	95% CI	%/Mean	95% CI
Depressive symptoms	2.20	(2.09, 2.31)	1.98	(1.85, 2.10)	2.60	(2.40, 2.79)
Anxiety symptoms	8.47	(8.36, 8.59)	8.29	(8.16, 8.43)	8.79	(8.57, 9.01)
Loneliness	4.78	(4.70, 4.87)	4.70	(4.60, 4.80)	4.93	(4.78, 5.08)
Age	67.77	(67.34, 68.20)	66.86	(66.34, 67.37)	69.36	(68.61, 70.11)
Sex						
Female	62.38	(60.12, 64.64)	67.65	(64.94, 70.36)	53.17	(49.24, 57.09)
Male	37.62	(35.36, 39.88)	32.35	(29.64, 35.06)	46.83	(42.91, 50.76)
Race/ethnicity						
Non-Hispanic White	84.32	(82.31, 86.33)	84.64	(82.18, 87.11)	83.75	(80.30, 87.20)
Non-Hispanic Black	6.35	(4.91, 7.80)	5.90	(4.17, 7.64)	7.14	(4.58, 9.70)
Hispanic or Latinx	3.80	(2.70, 4.91)	3.94	(2.57, 5.31)	3.57	(1.70, 5.44)
Non-Hispanic Other	5.53	(4.32, 6.73)	5.52	(4.00, 7.03)	5.54	(3.54, 7.54)
Educational attainment						
High school or less	13.65	(11.42, 15.88)	14.11	(11.29, 16.93)	12.84	(9.21, 16.47)
Some college or 2-year associate degree	27.31	(25.12, 29.51)	23.62	(21.05, 26.19)	33.76	(29.83, 37.69)
4-Year college or university degree	28.33	(26.51, 30.16)	30.18	(27.89, 32.47)	25.11	(22.10, 28.13)
Graduate degree	30.71	(29.00, 32.41)	32.09	(30.00, 34.22)	28.29	(25.45, 31.12)
Partnership status						
Coupled	64.36	(62.13, 66.58)	65.13	(62.39, 67.87)	63.01	(59.21, 66.80)
Not coupled	35.64	(33.42, 37.87)	34.87	(32.13, 37.61)	36.99	(33.20, 40.79)
Pre-COVID employment status						
Self-employed	7.60	(6.40, 8.80)	8.77	(7.09, 10.44)	5.56	(4.07, 7.05)
Employed full-time	21.33	(19.60, 23.07)	24.03	(21.79, 26.28)	16.62	(13.96, 19.28)
Employed part-time	9.43	(8.16, 10.69)	10.67	(9.06, 12.27)	7.26	(5.20, 9.32)
Unable	6.21	(4.91, 7.50)	2.83	(1.83, 3.83)	12.09	(9.10, 15.08)
Homemaker or family caregiver	3.02	(2.13, 3.91)	3.67	(2.41, 4.92)	1.89	(0.82, 2.96)
Unemployed and seeking work	1.51	(0.86, 2.17)	1.52	(0.07, 0.23)	1.50	(0.41, 2.59)
Retired	50.91	(48.66, 53.15)	48.51	(45.78, 51.25)	55.08	(51.19, 58.96)
Pre-COVID-19 social isolation						
High	27.69	(25.61, 29.76)	26.01	(23.50, 28.51)	30.61	(26.96, 34.26)
Low	72.31	(70.24, 74.39)	73.99	(71.49, 76.50)	69.39	(65.74, 73.04)
Previous diagnosis of depression (yes)	29.91	(27.36, 32.46)	26.69	(23.93, 29.45)	35.58	(31.06, 40.10)
Previous diagnosis of anxiety (yes)	23.10	(20.81, 25.39)	21.22	(18.66, 23.78)	26.41	(22.23, 30.60)
Use of any mobility aid (yes)	9.29	(7.84, 10.74)	4.36	(3.12, 5.60)	17.98	(14.75, 21.20)
Smoking status						
Never smoked	54.50	(52.24, 56.76)	58.92	(56.17, 61.67)	46.79	(42.93, 50.65)
Ex-smoker	38.80	(36.57, 41.02)	34.91	(32.23, 37.58)	45.58	(41.68, 49.48)
Current smoker	6.70	(5.39, 8.01)	6.17	(4.68, 7.67)	7.63	(5.17, 10.09)
Physical activity (one unit = 30 min)	4.00	(3.90, 4.09)	4.29	(4.17, 4.40)	3.49	(3.32, 3.66)
Alcohol consumption (no. of drinks)	2.86	(2.70, 3.02)	3.10	(2.91, 3.29)	2.44	(2.17, 2.71)

### Depressive Symptoms

**Figure 1.**
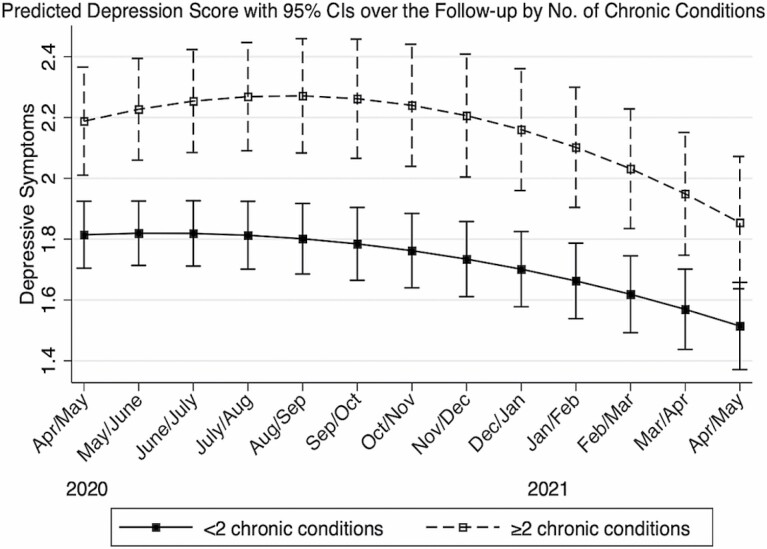
The trajectories of depressive symptoms according to baseline multimorbidity status, COVID-19 Coping Study, United States, April/May 2020–April/May 2021 (*N* = 4,024).

Multimorbidity was significantly associated with elevated depressive symptoms (β = 0.373; 95% CI: 0.158, 0.589), but not with the rate of change in depressive symptoms over time (Table 2). Regardless of multimorbidity status, the 12-month trajectories of depressive symptoms were not linear (Table 2, Figure 1). As shown in Figure 1, older adults with multimorbidity experienced persistently higher levels of depressive symptoms than those without multimorbidity and experienced a slight increase in symptoms from the baseline (April/May 2020) to the 6-month follow-up (September/October 2020). Both groups experienced a continuous decline in depressive symptoms from the 6th through 13th month of follow-up (April/May 2021; Figure 1). Details on estimates of covariates and random effects are presented in Supplementary Table 2.

**Table 2.** Population- and Attrition-Weighted, Multivariable-Adjusted Linear Mixed-Effects Models Estimating the Associations Between Baseline Multimorbidity and Participants’ Mental Health, COVID-19 Coping Study, United States, April/May 2020–April/May 2021 (*N* = 4,024)

Variable	Mental health outcomes					
	Depressive symptoms		Anxiety symptoms		Loneliness	
	β	95% CI	β	95% CI	β	95% CI
Intercept	3.100***	(1.860, 4.340)	9.729***	(8.139, 11.319)	5.875***	(4.944, 6.806)
Multimorbidity	0.373**	(0.158, 0.589)	0.385**	(0.150, 0.620)	0.096	(−0.080, 0.271)
Linear time	−0.059	(−0.145, 0.026)	−0.176***	(−0.256, −0.097)	0.026	(−0.001, 0.052)
Multimorbidity × Linear time	0.038	(−0.025, 0.100)	0.070*	(0.015, 0.125)	0.066**	(0.017, 0.115)
Quadratic time	−0.003*	(−0.005, −0.001)	−0.006***	(−0.008, −0.004)	−0.004***	(−0.005, −0.002)
Multimorbidity × Quadratic time	−0.003	(−0.008, 0.002)	−0.006**	(−0.011, −0.002)	−0.005*	(−0.008, −0.001)

*Notes:* Models are adjusted for age, sex, race/ethnicity, educational attainment, partnership status, pre-COVID employment status, pre-COVID degree of social isolation, previous diagnoses of depression and anxiety, use of any mobility aid, smoking status, physical activity, and alcohol consumption. Depressive symptoms were measured using the 8-item Center for Epidemiologic Studies Depression Scale, ranging from 0 to 8. Anxiety symptoms were measured using the 5-item Beck Anxiety Scale, ranging from 4 to 20. Loneliness was measured using the 3-item UCLA Loneliness Scale, ranging from 3 to 9.

***p < .0001, **p < .001, *p < .05.

### Anxiety Symptoms

**Figure 2.**
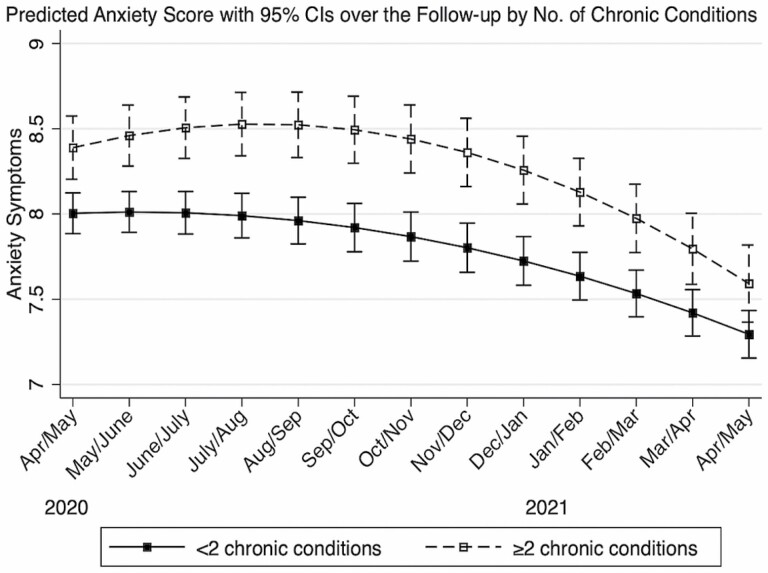
The trajectories of anxiety symptoms according to baseline multimorbidity status, COVID-19 Coping Study, United States, April/May 2020–April/May 2021 (*N* = 4,024).

Multimorbidity was significantly associated with elevated anxiety symptoms at baseline (β = 0.385; 95% CI: 0.150, 0.620), as well as rate of change in anxiety symptoms over time (Table 2). Regardless of multimorbidity status, the 12-month trajectories of anxiety symptoms were not linear (Table 2, Figure 2). Older adults with multimorbidity experienced persistently higher levels of anxiety symptoms than those without multimorbidity (Figure 2). Overall, both groups experienced a decrease in anxiety symptoms over the follow-up, but this rate of decrease was slower for older adults with multimorbidity (older adults without multimorbidity: β = −0.176; 95% CI: −0.256, −0.097; difference for those with multimorbidity: β = 0.070; 95% CI: 0.015, 0.125; Table 2). Details on estimates of covariates and random effects are presented in Supplementary Table 2.

### Loneliness

**Figure 3.**
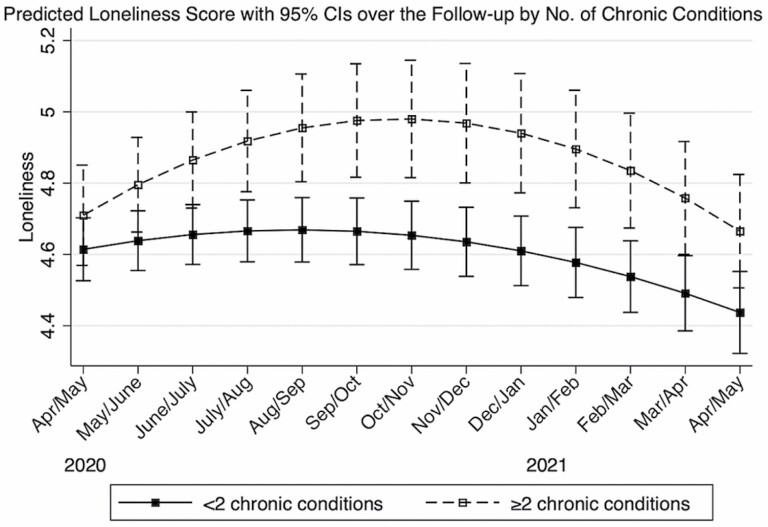
The trajectories of loneliness according to baseline multimorbidity status, COVID-19 Coping Study, United States, April/May 2020–April/May 2021 (*N* = 4,024).

Multimorbidity was not associated with loneliness at baseline (Table 2). However, multimorbidity was associated with the rate of change in loneliness over time (Table 2, Figure 3). Regardless of multimorbidity status, the 12-month trajectories of loneliness were not linear (Table 2, Figure 3). Older adults with multimorbidity reported increasing loneliness from baseline through the sixth month of follow-up (September/October 2020), whereas loneliness decreased in both groups from the sixth through thirteenth month of follow-up (April/May 2021; Table 2, Figure 2). Loneliness remained persistently higher among those with multimorbidity at the pandemic onset across the entire follow-up period (Figure 2) Details on estimates of covariates and random effects are presented in Supplementary Table 2. Additional analyses comparing three mental health conditions between those without chronic conditions and those with one or more conditions showed similar result patterns and are available upon request.

## Discussion

In this longitudinal study of older adults in the United States throughout the COVID-19 pandemic, multimorbidity at the pandemic onset was associated with elevated depressive and anxiety symptoms at baseline in April/May 2020. Throughout the subsequent 12 months, those with multimorbidity had persistently higher depressive symptoms, anxiety symptoms, and loneliness than those without multimorbidity. Moreover, those with multimorbidity experienced faster rates of change in anxiety symptoms and loneliness, but not depressive symptoms throughout the pandemic. Overall, the findings underscore the unique and persistent mental health risks experienced by older adults with multimorbidity during the COVID-19 pandemic.

The mental health trajectories observed in this study are comparable to several longitudinal observational studies focusing on American and British adults, with an average five-month follow-up time in the early months of the COVID-19 pandemic (e.g., March/April–August 2020; Daly et al., 2020; Fancourt et al., 2021; Fluharty et al., 2021; Niedzwiedz et al., 2020; Riehm et al., 2021). Most studies have identified a peak in symptoms of depression, anxiety, and loneliness at the onset of the pandemic (March–April 2020; Daly et al., 2020; Fancourt et al., 2021; Fluharty et al., 2021; Niedzwiedz et al., 2020; O’Connor et al., 2021; Riehm et al., 2021), followed by a decrease in symptoms over time (Daly et al., 2020; Fancourt et al., 2021; Fluharty et al., 2021; Riehm et al., 2021). Different from the turning points identified in these previous studies, we observed worsening symptoms over the first 6 months of the pandemic with a peak in mental health symptoms in September/October 2020, followed by improvement in symptoms over the subsequent six months.

The improvement in symptoms over time may indicate that older adults began mentally adapting to the ongoing pandemic through coping strategies (Finlay et al., 2021; Fuller & Huseth-Zosel, 2021; McElroy-Heltzel et al., 2022; Pearman et al., 2021). In a qualitative study among COVID-19 Coping Study cohort (*N* = 5,180), Finlay et al. (2021) found that older adults employed both cognitive and behavioral strategies that included problem-focused, emotion-focused, and social support coping during the early months of the pandemic. Several other studies have identified proactive coping behaviors such as maintaining a daily routine, engaging in outdoor activities, keeping a positive mindset, forming social connections, and taking COVID-19 precautions that operated to relieve mental health symptoms (Finlay et al., 2021; Fuller & Huseth-Zosel, 2021; Pearman et al., 2021). Individuals who were proactively coping since the onset of the pandemic were found to have better mental health as lockdowns were introduced, as well as faster recovery from mental health symptoms over time (Fluharty et al., 2021; Pearman et al., 2021). Beyond coping at the individual level, improvements in mental health also overlapped with the introduction and widespread distribution of COVID-19 vaccines. By May 22, 2021, 57% of U.S. adults had received at least one vaccine dose and the coverage was highest among persons aged 65 and older (80%; Diesel et al., 2021). Older adults and individuals with multiple chronic conditions who were among the first in line to receive a vaccine may share a general sense of hopefulness, thereby reducing psychological distress such as depressive mood and anxiety.

Of note, our findings highlight that pre-pandemic mental health disparities continue to disproportionately affect older adults with multimorbidity during the COVID-19 pandemic. Older adults with multimorbidity in this study reported worse depressive and anxiety symptoms at the pandemic onset and persistently elevated symptoms of depression, anxiety, and loneliness throughout the 12-month follow-up than those without multimorbidity. Fear associated with higher susceptibility to COVID-19 infection and its related complications could induce more COVID-19-related worries and stresses, which may increase the risk of elevated depressive and anxiety symptoms (Davis et al., 2021; Pearman et al., 2021). Moreover, worry and stress related to illness and delayed health care during the pandemic could also contribute to worsening mental health symptoms. During the pandemic, widespread media coverage highlighted that COVID-19 was more severe for those with an older age, who had chronic diseases, and comorbidities. This media coverage added onto widespread ageism and ableism in the public discourse, which portrays older adults with comorbidity as vulnerable, frail, and burdensome on society (Ayalon et al., 2021). As a result, older adults with comorbidity may experience stronger internal and external ageism (Morrow-Howell et al., 2020). This may have lasting mental health impacts. Research has shown that older adults with more negative self-perceptions of aging were more likely to report higher psychological distress and loneliness during the lockdown (Losada-Baltar et al., 2021). In addition, prior studies have found that functional limitations, disability, and self-perceptions of health may explain the associations between chronic physical health conditions and symptoms of depression and loneliness (Gunn et al., 2012; Parajuli et al., 2021; Stegmann et al., 2010; Stickley & Koyanagi, 2018). Similar mechanisms may link multimorbidity to elevated anxiety symptoms. Decrements in normal activities; increases in pain, symptom burden, and sleep problems; and increase in social and physical isolation—which are common experiences of living with multimorbidity—are likely to induce psychological distress (Backe et al., 2018; de Ridder et al., 2008; Sindi et al., 2020). Future research should further elucidate the mediating mechanisms linking multimorbidity to symptoms of depression, anxiety, and loneliness.

This study has several limitations. We employed nonprobability sampling strategies to recruit participants through word-of-mouth snowball sampling and health research databases such as NIH ResearchMatch. To address the potential biases related to a nonrandom sample, we generated population weights based on nationally representative data from the 2018 American Community Survey and applied population weights to all analyses. However, there may be residual bias if there are any unmeasured factors that influenced selection into the study, the study exposure, and outcome variables that were uncorrelated with the sociodemographic factors used to generate the weights. Our results may not be generalizable to those who do not use internet or mobile data, which may include people with limited technology literacy and limited access to internet or smartphones. We defined multimorbidity using seven disease counts and did not account for the symptoms or severity of these health conditions. Although we asked about the presence of other limiting, longstanding health conditions, we could not capture all possible chronic conditions that may contribute to multimorbidity, so our study may underestimate the prevalence of multimorbidity (Boersma et al., 2020).

We dichotomized multimorbidity as <2 chronic conditions versus ≥2 chronic conditions. We were not able to test the trend in the count of chronic conditions in relation to mental health symptoms due to the insufficient number of respondents with four or more chronic conditions. As data collection began early in the COVID-19 pandemic (April/May 2020), we did not have pre-COVID data available on the mental health outcomes. It is unknown if the observed relationship between multimorbidity and mental health symptoms trajectories in this cohort would have been different had the COVID-19 pandemic not occurred. Finally, we need to be cautious about the potential bidirectional relationships between multimorbidity and symptoms of depression, anxiety, and loneliness (Qiao et al., 2022; Triolo et al., 2020). Future research is needed to assess the long-term effects of COVID-19-related psychological distress on the onset and progression of multimorbidity over time, in addition to the long-term mental health outcomes of middle-aged and older adults who entered the pandemic with multimorbid health conditions.

Despite the limitations, our findings are robust and noteworthy. To the best of our knowledge, this study is among the first to examine longitudinal mental health trajectories in relation to multimorbidity in the context of the COVID-19 pandemic among middle-aged and older U.S. adults. We used 13 monthly waves of data from April/May 2020 through April/May 2021, providing timely and fine-grained evidence on the longitudinal changes in depressive symptoms, anxiety symptoms, and loneliness among U.S. older adults amidst several waves of the COVID-19 pandemic. We had rich covariate data, including pre-COVID-19 social and economic factors and mental health diagnoses. Our sample size was large and covered all 50 U.S. states and the District of Columbia. By incorporating population sampling and attrition weights, we were able to minimize potential sampling biases and improve the generalizability of findings. We used measures of depressive symptoms, anxiety symptoms, and loneliness that have been validated in older adults and are commonly used in aging research, including in the U.S. Health and Retirement Study and its International Partner Studies of aging, enhancing the comparability of our findings to existing research.

This study suggests that public health efforts would be beneficial to help promote mental health resilience among middle-aged and older adults with multimorbidity during times of public health crisis, such as potential future pandemic events. Middle-aged and older adults with multiple chronic conditions may benefit from tailored interventions that focus on resilience and coping skills, as well as support for chronic disease management and reduction of infection risk during pandemics. Along with routine visits of chronic care management in primary care and home care settings, enhanced mental health screening in older adults would facilitate the diagnosis and treatment of potential mental health conditions. Primary and specialty care physicians should be encouraged to maintain care for their patients with multimorbidity and to quickly respond to their changing needs resulting from disrupted routine checkups, delays in care, or pandemic safety measures. As telemedicine for chronic care management has been widely adopted since the pandemic, it is pressing to ensure timely and effective access to virtual health services for individuals with multiple chronic conditions, with a priority to address digital utilization disparities among aging populations.

## Supplementary Material

igac047_suppl_Supplementary_Material
