# Supplementary material for: Multimorbidity and Mental Health Trajectories Among Middle-Aged and Older U.S. Adults During the COVID-19 Pandemic: Longitudinal Findings From the COVID-19 Coping Study
Source: Innov Aging. 2022 Jul 30;6(5):igac047. doi: 10.1093/geroni/igac047 (PMC9403728; doi:10.1093/geroni/igac047)
Supplement: igac047_suppl_Supplementary_Material [file igac047_suppl_supplementary_material.docx]

Supplementary Table 1. Population-weighted prevalence of multimorbidity, counts and types of chronic conditions, COVID-19 Coping Study, United States, April/May 2020, (N=4,024)

|  | Prevalence (%) | 95% CI |
| --- | --- | --- |
| Multimorbidity | 36.44 | (34.24, 38.65) |
| Counts of chronic conditions |  |  |
| 0 | 29.36 | (27.36, 31.35) |
| 1 | 34.20 | (32.08, 36.31) |
| 2 | 20.37 | (18.56, 22.17) |
| 3 | 11.59 | (10.00, 13.18) |
| 4 | 3.28 | (2.44, 4.12) |
| 5-7 | 1.20 | (--, --)* |
| Types of chronic conditions |  |  |
| Hypertension | 47.66 | (45.41, 49.91) |
| Diabetes | 14.11 | (12.41, 15.81) |
| Heart disease | 11.40 | (9.91, 12.89) |
| Asthma | 11.57 | (10.28, 12.87) |
| Chronic obstructive pulmonary disease | 6.46 | (5.22, 7.70) |
| Cancer | 15.90 | (14.30, 17.50) |
| Other limiting, long-standing health condition | 21.91 | (20.00, 23.84) |

*Note.* *The 95% confidence interval cannot be estimated due to insufficient sample size.

Supplementary Table 2. Population-and attrition-weighted, multivariable-adjusted linear mixed-effects models estimating the associations between baseline multimorbidity and participants’ mental health, COVID-19 Coping Study, United States, April/May 2020- April/May 2021 (N=4,024)

| Variable | Mental health outcomes | | | | | |
| --- | --- | --- | --- | --- | --- | --- |
|  | Depressive symptoms | | Anxiety symptoms | | Loneliness | |
|  | $\beta$ | 95% CI | $\beta$ | 95% CI | $\beta$ | 95% CI |
| Intercept | 3.100*** | (1.860, 4.340) | 9.729*** | (8.139, 11.319) | 5.875*** | (4.944, 6.806) |
| Multimorbidity | 0.373** | (0.158, 0.589) | 0.385** | (0.150, 0.620) | 0.096 | (-0.080, 0.271) |
| Linear time | -0.059 | (-0.145, 0.026) | -0.176*** | (-0.256, -0.097) | 0.026 | (-0.001, 0.052) |
| Multimorbidity x Linear time | 0.038 | (-0.025, 0.100) | 0.070* | (0.015, 0.125) | 0.066** | (0.017, 0.115) |
| Quadratic time | -0.003* | (-0.005, -0.001) | -0.006*** | (-0.008, -0.004) | -0.004*** | (-0.005, -0.002) |
| Multimorbidity x Quadratic time | -0.003 | (-0.008, 0.002) | -0.006** | (-0.011, -0.002) | -0.005* | (-0.008, -0.001) |
| Age | -0.026** | (-0.42, -0.010) | -0.040*** | (-0.060, -0.019) | -0.015* | (-0.026, -0.003) |
| Female (ref: male) | 0.472*** | (0.256, 0.689)*** | 0.637*** | (0.402, 0.871) | 0.233** | (0.064, 0.403) |
| Race/Ethnicity (Non-Hispanic White) |  |  |  |  |  |  |
| Non-Hispanic Black | -0.393 | (-0.910, 0.124) | 0.084 | (-0.350, 0.518) | -0.122 | (-0.537, 0.293) |
| Hispanic or Latinx | -0.162 | (-0.810, 0.487) | 0.087 | (-0.603, 0.777) | -0.092 | (-0.603, 0.420) |
| Non-Hispanic Other | 0.149 | (-0.366, 0.665) | 0.303 | (-0.422, 1.029) | -0.192 | (-0.559, 0.176) |
| Education attainment (ref: Graduate degree) |  |  |  |  |  |  |
| High school or less | 0.397 | (-0.076, 0.870) | 0.051 | (-0.408, 0.509) | 0.041 | (-0.272, 0.355) |
| Some college or 2-year associate degree | 0.179 | (-0.058, 0.417) | 0.101 | (-0.149, 0.351) | 0.059 | (-0.130, 0.248) |
| 4-year college or university degree | 0.031 | (-0.123, 0.184) | -0.082 | (-0.263, 0.098) | -0.040 | (-0.172, 0.091) |
| Coupled (ref: Not coupled) | -0.370** | (-0.616, -0.125) | 0.047 | (-0.205, 0.300) | -0.828*** | (-1.018, -0.638) |
| Pre-COVID employment status (ref: retired) |  |  |  |  |  |  |
| Self-employed | 0.272 | (-0.060, 0.605) | 0.159 | (-0.275, 0.592) | 0.021 | (-0.255, 0.297) |
| Employed full-time | 0.184 | (-0.744, 0.442) | 0.132 | (-0.155, 0.419) | 0.083 | (-0.118, 0.285) |
| Employed part-time | 0.313 | (-0.039, 0.666) | 0.084 | (-0.194, 0.362) | -0.001 | (-0.232, 0.230) |
| Unable | 0.865 | (0.279, 1.451) | 0.975** | (0.329, 1.622) | 0.339 | (-0.156, 0.834) |
| Homemaker or family caregiver | 0.477 | (-0.218, 1.171) | 0.687 | (-0.80, 1.453) | -0.064 | (-0.529, 0.400) |
| Unemployment and seeking work | 0.207 | (-0.355, 0.768) | -0.018 | (-0.738, 0.702) | -0.462 | (-1.079, 0.154) |
| High degree of social isolation (ref: low) | 0.208 | (-0.041, 0.457) | -0.086 | (-0.335, 0.162) | 0.194* | (0.001, 0.386) |
| Previous diagnosis of depression | 1.173*** | (0.870, 1.475) | 0.626*** | (0.333, 0.918) | 0.500*** | (0.268, 0.725) |
| Previous diagnosis of anxiety | 0.651** | (0.286, 1.015) | 1.247*** | (0.905, 1.590) | 0.347** | (0.090, 0.603) |
| Use of mobility aid | 0.584** | (0.166, 1.001) | 0.847*** | (0.421, 1.273) | 0.239 | (-0.087, 0.565) |
| Smoking status (ref: Never smoked) |  |  |  |  |  |  |
| Ex-smoker | 0.125 | (-0.077, 0.327) | 0.184 | (-0.040, 0.408) | 0.160* | (0.005, 0.316) |
| Current smoker | 0.494* | (0.028, 0.959) | 0.625** | (0.190, 1.060) | 0.307 | (-0.113, 0.727) |
| Physical activity (one unit=30 mins) | -0.088** | (-0.140, -0.035) | -0.012 | (-0.066, 0.041) | -0.038 | (-0.080, 0.004) |
| Alcohol consumption (No. of drinks) | -0.001 | (-0.030, 0.027) | -0.008 | (-0.040, 0.024) | -0.009 | (-0.031, 0.014) |
| Var(intercept) | 1.697*** | (1.619, 1.778) | 1.867*** | (1.776, 1.963) | 1.337*** | (1.284, 1.392) |
| Var(random slope—linear time) | 0.102*** | (0.090, 0.115) | 0.091*** | (0.080, 0.102) | 0.085*** | (0.077, 0.093) |
| Var(random error) | 1.240*** | (1.202, 1.280) | 1.288*** | (1.256, 1.322) | 0.930*** | (0.902, 0.959) |

*Note.* Depressive symptoms were measured using the 8-item CES-D Scale, ranging from 0-8. Anxiety symptoms were measured using the 5-item Beck Anxiety Scale, ranging from 4-20. Loneliness was measured using the 3-item UCLA Loneliness Scale, ranging from 3-9.

*** p<.0001, **p<.001, *p<.05.
